# *Plasmodium* prevalence and artemisinin-resistant falciparum malaria in Preah Vihear Province, Cambodia: a cross-sectional population-based study

**DOI:** 10.1186/1475-2875-13-394

**Published:** 2014-10-06

**Authors:** Philippe Bosman, Jorgen Stassijns, Fabienne Nackers, Lydie Canier, Nimol Kim, Saorin Khim, Sweet C Alipon, Meng Chuor Char, Nguon Chea, Lek Dysoley, Rafael Van den Bergh, William Etienne, Martin De Smet, Didier Ménard, Jean-Marie Kindermans

**Affiliations:** Médecins Sans Frontières, Duprestreet 94, 1090 Brussels, Belgium; Epicentre, Saint Sabin Street 8, 75011 Paris, France; Institut Pasteur of Cambodia, Malaria Molecular Epidemiology Unit, 5 Boulevard Monivong, PO Box 983, Phnom Penh, Cambodia; Médecins Sans Frontières, #19 Street 388, Tuol Svay Prey I, Phnom Penh, Chamkarmon Cambodia; National Center for Parasitology, Entomology and Malaria Control (CNM), Ministry of Health (MoH), Corner St 92, Village Trapaing Svay, Sangkat Phnom Penh Thmey, Khan Sen Sok Phnom Penh, Cambodia

**Keywords:** Malaria, *Plasmodium falciparum*, Epidemiology, Artemisinin resistance, Polymerase chain reaction, K13-Propeller, Cambodia

## Abstract

**Background:**

Intensified efforts are urgently needed to contain and eliminate artemisinin-resistant *Plasmodium falciparum* in the Greater Mekong subregion. Médecins Sans Frontières plans to support the Ministry of Health in eliminating *P. falciparum* in an area with artemisinin resistance in the north-east of Cambodia. As a first step, the prevalence of *Plasmodium* spp. and the presence of mutations associated with artemisinin resistance were evaluated in two districts of Preah Vihear Province.

**Methods:**

A cross-sectional population-based study using a two-stage cluster sampling was conducted in the rural districts of Chhaeb and Chey Saen, from September to October 2013. In each district, 30 clusters of 10 households were randomly selected. In total, blood samples were collected for 1,275 participants in Chhaeb and 1,224 in Chey Saen. Prevalence of *Plasmodium* spp. was assessed by PCR on dried blood spots. *Plasmodium falciparum* positive samples were screened for mutations in the K13-propeller domain gene *(PF3D7_1343700)*.

**Result:**

The prevalence of *Plasmodium* spp. was estimated at 1.49% (95% CI 0.71–3.11%) in Chhaeb and 2.61% (95% CI 1.45–4.66%) in Chey Saen. Twenty-seven samples were positive for *P. falciparum*, giving a prevalence of 0.16% (95% CI 0.04–0.65) in Chhaeb and 2.04% (95% CI 1.04–3.99%) in Chey Saen. Only 4.0% of the participants testing positive presented with fever or history of fever. K13-propeller domain mutant type alleles (C580Y and Y493H) were found, only in Chey Saen district, in seven out of 11 *P. falciparum* positive samples with enough genetic material to allow testing.

**Conclusion:**

The overall prevalence of *P. falciparum* was low in both districts but parasites presenting mutations in the K13-propeller domain gene, strongly associated with artemisinin-resistance, are circulating in Chey Saen.The prevalence might be underestimated because of the absentees – mainly forest workers - and the workers of private companies who were not included in the study. These results confirm the need to urgently develop and implement targeted interventions to contain and eliminate *P. falciparum* malaria in this district before it spreads to other areas.

## Background

Over the last decade, huge efforts have been made to control malaria. It is estimated that mortality rates attributable to malaria have decreased by 42% worldwide and the incidence of malaria by 25% [1]. Several countries are now classified by the World Health Organization (WHO) as being in the malaria pre-elimination or elimination phase. Such declining trends have been observed in the Greater Mekong subregion [2], including Cambodia. Unfortunately, these achievements are threatened by the emergence of artemisinin-resistant parasites in the region [3–5], initially detected in Pailin, Cambodia, near to the Thai-Cambodian border [6]. In 2009, in response to this major public health concern, the Thai and Cambodian governments, with the support of the WHO, implemented a strategy to contain artemisinin resistance and to protect the efficacy of artemisinin-based combination therapy (ACT). The strategy was based on a wide range of activities, including large scale distributions of long-lasting insecticide-treated nets (LLIN), the implementation of accurate and widely-available malaria rapid diagnostic tests (RDT), the ban of artemisinin monotherapies and the universal access to ACT [7].

Preah Vihear Province, located in the north of Cambodia, sharing a border with Lao People’s Democratic Republic (Champasak Province) and Thailand (Sisaket and Ubon-Ratchathani Provinces), was recently considered at risk for artemisinin resistance. In 2007, a study was conducted in three Cambodian Provinces and, in Preah Vihear, the prevalence of *Plasmodium falciparum* infections was estimated at 9.9% through microscopy; 0.7% were mixed infections [8]. The substantial scaling-up of malaria control strategies has considerably contributed to reduce the burden of malaria, though there are no recent reliable prevalence figures for the district. Data collected by the network of Village Malaria Workers (the Malaria Information System, also known as the Village Malaria Worker Database or D0 system) are unlikely to reflect the transmission at community level and properly assess the actual parasite reservoir. Home-based treatment or treatment from the private health sector remain the first choice of many patients with fever [9]. Furthermore, considering that many infections are asymptomatic, figures reported through surveillance systems underestimate the true burden of malaria infections. Symptomatic infections may contribute considerably to transmission, including in areas of low transmission intensity [10], and thus monitoring of symptomatic cases only (passive case detection) represents a poor indicator for progress towards elimination. Most subclinical infections, especially with low parasite densities, would be missed by microscopy or RDTs and their detection requires molecular techniques with higher sensitivity [11, 12].

Médecins Sans Frontières (MSF) decided to support the Ministry of Health in eliminating *P. falciparum* in Preah Vihear Province. As a first step, the prevalence of *Plasmodium* spp. and *P. falciparum* carriage at population level was assessed in two districts of Preah Vihear Province, by using a molecular detection method capable of detecting submicroscopic malaria infections. Each *P. falciparum* positive sample was screened for mutations in the K13-propeller domain gene, associated with artemisinin-resistance [13].

## Methods

### Study area

**Figure 1:**
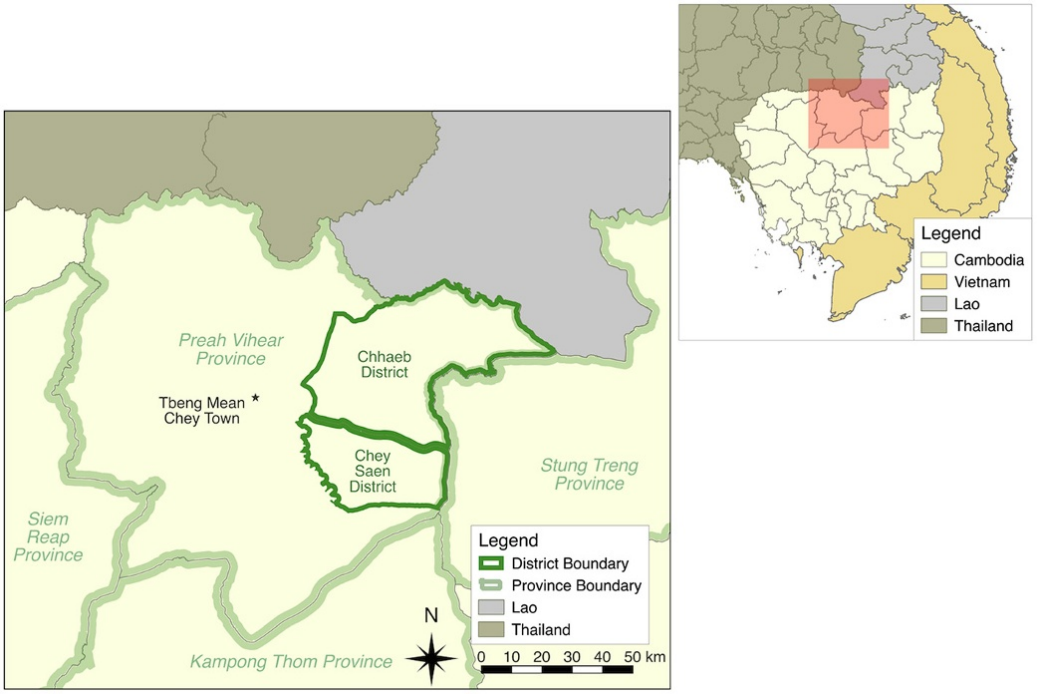
**Map of Cambodia, Preah Vihear Province, Chhaeb and Chey Saen districts.**

The study was conducted from September to October 2013, at the end of the rainy season, in two neighbouring districts, Chhaeb and Chey Saen, located in the Northern Province of Preah Vihear, Cambodia (Figure 1). These rural districts are located at an altitude of 100 metres above sea level and their landscape alternates between forestry and cultivated areas. Chhaeb district covers an area of 2,500 km^2^ and has a population of approximately 18,455 inhabitants residing in 26 villages (according to the National Cambodian Commune Database, based on the 2008 General Population Census [14]. Chey Saen district covers 1,100 km^2^ with a population of 21,407 inhabitants living in 21 villages. The climate is tropical with a wet monsoon season extending from mid-May to early October. Malaria transmission is seasonal, with a peak occurring usually in August and September.

### Design and participants

To estimate the prevalence of *Plasmodium* spp in the population, a cross-sectional community-based study was conducted in each district using a two-stage random cluster sampling method. In each district, 30 clusters were selected with probability proportional to the population size of their villages. In each village including at least one cluster, a list of households, including residents, migrants and seasonal workers, was drawn up in collaboration with the village authorities, who provided the household lists. Temporary workers living in the compound of private companies were not included, due to possible sensitivities with regards to this population. In each cluster, 10 households were selected by systematic random sampling in the village households list. For every selected household, all members aged six months and older were invited to participate in the study. The study was done in coordination with the local authorities and prior to the survey, information and sensitization campaigns were organized for the communities. To minimize absenteeism, participants were given an incentive for their participation (a piece of local fabric, krama).

### Data collection

For each participant, the age, sex, status (permanent resident, visitor or seasonal worker), use of mosquito net the night before the interview were recorded. History of fever in the last 24 hours previous to the interview and body temperature of individuals complaining of fever at the time of the survey or of history of fever within the previous 24 hours were collected as well. The bed nets owned by the household (found in the household at the time of the study) were observed and counted.

### Sample collection

For each participant aged six months and older, capillary blood was collected by finger prick. Five microlitres (μl) were transferred to a 96-wells PCR plate pre-filled with a 5 mm punch of filter paper, as previously described [15, 16]. Blood specimens were brought within 7 hours to the mobile laboratory of the Institut Pasteur of Cambodia (IPC) [16] located in Tbeng Mean Chey, capital of the province. Individuals with axillary temperature ≥ 37.5°C or reporting fever in the last 24 hours were tested in parallel for malaria by RDT (SD Bioline Ag P.f./Pan; Standard Diagnostics Ref 05FK60, Inc: Suwon City, Republic of Korea).

### Laboratory procedures

#### DNA extraction

Blood spots in 96-well plates were lysed overnight in saponin solution and washed. DNA extraction was performed with Instagene® Matrix resin (Bio-Rad, France), according to the protocol adapted from the supplier’s recommendations [15].

#### RT-PCR detection of Plasmodium

*Plasmodium* DNA was detected using a qualitative Real-Time PCR (RT-PCR) assay targeting the *Plasmodium* cytochrome b gene [15]. Raw data were emailed for validation to IPC in Phnom Penh. Results were available within 30 hours of blood collection, allowing to contact the participants with positive results and to provide free treatment within two days after the initial blood sampling.

#### Species differentiation

For all samples positive for *Plasmodium* spp., species differentiation was done in Phnom Penh using RT- PCR assays amplifying *P. falciparum*, *Plasmodium vivax (*and *Plasmodium knowlesi)*, *Plasmodium ovale* and *Plasmodium malariae* [16].

#### Mutations in the K13-propeller domain gene (PF3D7_1343700)

*P. falciparum* positive samples were screened for mutations in the K13-propeller domain gene [13], a molecular marker associated with artemisinin resistance, according to the procedure described on the Nature protocols website [17].

The K13-propeller domain gene was amplified using nested PCR. PCR products were detected using 2% agarose gel electrophoresis and ethidium bromide staining. Double-strand sequencing of PCR products was performed by Macrogen (Korea). Electrophoregrams were visualized and analysed with CEQ2000 genetic analysis system software (Beckman Coulter, Villepinte, France). The amino acid sequences were compared with the wild-type amino acid sequences (GenBank accession number, XM_001350122). The presence of single nucleotide polymorphisms (SNPs) was confirmed by reading both the forward and the reverse strands. Parasites with mixed alleles (in which both wild-type and mutant alleles were present) were considered mutants for estimation of the prevalence of the SNPs.

#### Quality controls

In each screening run, controls assessing both the DNA extraction and the PCR steps were included: one positive extraction control (*P. falciparum* 3D7 at 500 parasites/μl) and one negative extraction control (with negative blood) both extracted at the same time as the samples; two PCR positive controls (*P. falciparum* DNA extracts at 1000 parasites/μl and at 1 parasite/μl) and one PCR negative control (water). In each PCR run for *Plasmodium* species, one positive plasmid control containing targeted DNA fragments specific to each species (*P. falciparum, P. vivax, P. malariae* and *P. ovale*), and two PCR negative controls were included.

### Treatment of positive cases

Participants with a positive malaria RDT or positive by PCR were offered free treatment according to national guidelines: a three-day course of dihydroartemisinin/piperaquine (DHA-PIP) or quinine for seven days in case of women in the first trimester of pregnancy. Carriers of *P. falciparum* with a mutant-type allele were traced and offered treatment with atovaquone and proguanil.

### Sample size and statistical analysis

To detect a 5% prevalence of *Plasmodium* carriage with a precision of 2%, accounting for 20% possible non-respondents (refusals or absentees) and blood sampling failure, a sample of 1,140 individuals was required in each district (α = 0.05, design effect = 2). Considering a mean household size of 4 as indicated in the 2010 DHS survey conducted in Cambodia, the minimum sample size was 285 households per district, which represents 10 households in each of the 30 clusters. Data were double entered in Epi-Info 3.5.4 and transferred to Stata 11 (StataCorp LP, College Station, TX USA) for data cleaning and analysis. Analyses were adjusted for the cluster study design. Proportions are presented with their 95% confidence intervals (95% CI) and design effect (DE).

### Ethics

The study was approved by the Ethics Review Board of Médecins Sans Frontières and by the Cambodian National Ethics Committee on Health Research (NECHR, approved 24th June 2013, number 0094NECHR). Written informed consent was obtained from each participant or legal representative for children aged less than 18 years old before participation.

## Results

### Characteristics of study population

Out of 600 households selected, 577 were present on the day of the study and accepted to participate, 292 in Chhaeb and 285 in Chey Saen district, representing 1,376 inhabitants in Chhaeb and 1,296 in Chey Saen (average household size of 4.6 persons). Of the 2,651 household members aged six months or more (respectively 1,366 in Chhaeb and 1,285 in Chey Saen), 13 refused the blood sampling (anecdotally reported as usually resulting from a fear of being pricked) and 139 absentees could not be sampled after two visits. Half of the absentees were men aged 15 to 34 years and most were reported being in the rice field or in the forest for several days. The majority of the participants were residents, with only 14 seasonal workers residing in other Cambodian Provinces and 13 visitors. In both districts, 53% of the participants were female and about 10% were younger than five years (Table 1). Except for a slight over-representation of the group aged 5–14 years in Chhaeb, the age and sex distribution of both sampled populations accorded well with the results from the Cambodian Census 2008.

**Table 1:** **Characteristics of study participants, Chhaeb and Chey Saen districts, September – October 2013, Cambodia**

	Chhaeb	Chey Saen
Villages [n]	22	18
Households [n]	292	285
Household size [Mean (range)]	4.7 (1–10)	4.5 (1–10)
Eligible participants < 6 months [n]	1,366	1,285
Blood sample collected [n (%)]	1,275 (93.3)	1,224 (95.3)
Age groups (years) [n (%)]		
< 5	149 (11.7)	125 (10.2)
5 – 14	386 (30.3)	310 (25.3)
15 – 24	243 (19.1)	279 (22.8)
25 – 34	175 (13.7)	191 (15.6)
35 – 44	121 (9.5)	126 (10.3)
≥ 45	201 (15.8)	193 (15.8)
Female n (%)	677 (53.1)	653 (53.3)

### Quality controls results

**Figure 2:**
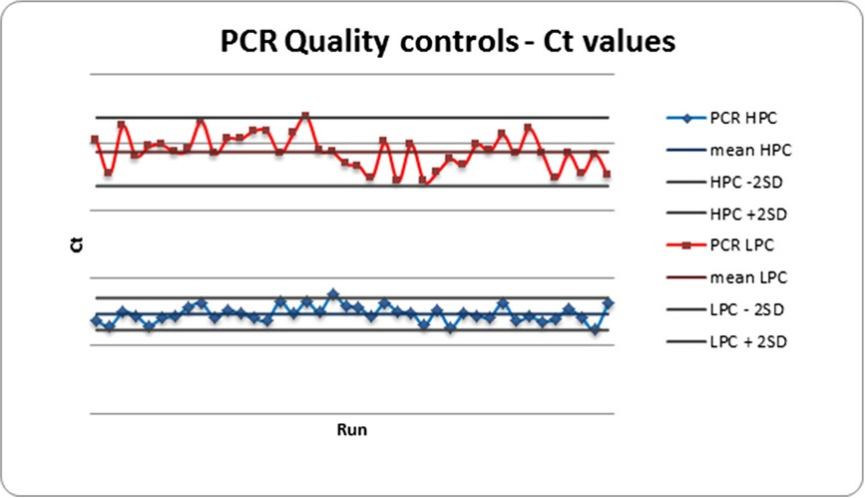
**X-chart of Ct values of PCR quality controls.** *X-axis: Cycle threshold (Ct); Y axis: series of 40 PCR runs; HPC: high positive control; LPC: low positive control; SD: standard deviation*.

PCR runs were validated based on the performance of quality controls. A total of 276 quality controls were done, representing 10% of the test performed. Among the 40 PCR screening runs performed during the study, six runs showed invalid quality control results and were repeated on the same day. Repeated runs were validated. Ct (cycle threshold) values of PCR controls were plotted on an X-chart (Figure 2) demonstrating the good reproducibility of the assay.

### Prevalence of *Plasmodium*and species differentiation

In Chhaeb District, 19 out of 1,275 participants were screened positive for *Plasmodium* spp. (Table 2), giving a prevalence of 1.49% (95% CI: 0.71 – 3.11%; DE: 2.53) – only one of these had fever (body temperature > 37.5°C). Almost two thirds (12/19) of *Plasmodium* carriers identified were male. *P. falciparum* was found in two adult females, giving a proportion of 10.5% of *P. falciparum* (2/19) and an overall prevalence of *P. falciparum* of 0.16% (95% CI: 0.04 – 0.65%; DE 0.98). Other *Plasmodium* infections (n = 17) were due to *P. vivax* (prevalence: 1.33%; 95% CI: 0.59 – 2.98%; DE 2.70). *P. ovale, P. malariae,* and mixed infections were not detected.

**Table 2:** ***Plasmodium*** **carriage detected by real-time PCR and species differentiation per age and sex, Chhaeb and Chey Saen districts, Cambodia, 2013**

N	Chhaeb						Chey Saen*					
	Male			Female			Male			Female		
Age (years)	All	***Pf p***ositive	***Pv***positive	All	***Pf p***ositive	***Pv***positive	All	***Pf p***ositive	***Pv***positive	All	***Pf p***ositive	***Pv***positive
< 5	75	0	0	74	0	1	58	0	0	67	0	0
5-14	198	0	3	188	0	0	156	3	0	154	2	0
15-24	107	0	3	136	0	2	121	5	1	158	3	2
25-34	75	0	4	100	0	0	92	3	1	99	2	0
35-44	58	0	2	63	1	1	58	4	1	68	0	0
≥45	85	0	0	116	1	1	86	2	0	107	1	1
Total	598	0	12	677	2	5	571	17	3	653	8	3

*Pf : Plasmodium falciparum; Pv: Plasmodium vivax;* *One participant positive for *Plasmodium malariae.*

In Chey Saen district, 32 out of 1,224 participants tested positive for *Plasmodium* spp. (Table 2), giving a prevalence of 2.61% (95% CI: 1.45 – 4.66%; DE: 2.68) with a majority of *P. falciparum* infections (n = 25; proportion of *P. falciparum* = 78.1%); only one case presented with fever (body temperature > 37.5°C). One third (8/25) of *P. falciparum* carriers were female. The overall prevalence of *P. falciparum* was 2.04% (95% CI: 1.04 – 3.99%; DE 2.78). Six infections were due to *P. vivax* (prevalence: 0.49%; 95% CI: 0.20 – 1.19%; DE 1.14). *Plasmodium malariae* was found in one participant. *Plasmodium ovale* and mixed infections were not detected.

In both districts, *Plasmodium* spp. carriers were found in all age groups except in children younger than five years. Out of the two *Plasmodium* spp carriers presenting with fever, one had a malaria RDT positive for non-*P. falciparum* species that was later confirmed as *P. vivax*; the other had a negative malaria RDT confirmed afterwards as *P. falciparum*. Other *Plasmodium* spp carriers (49/51; 96.0%) did not complain about fever on enrollment or in the preceding 24 hours.

### Prevalence of K13-propeller mutant-type alleles

**Figure 3:**
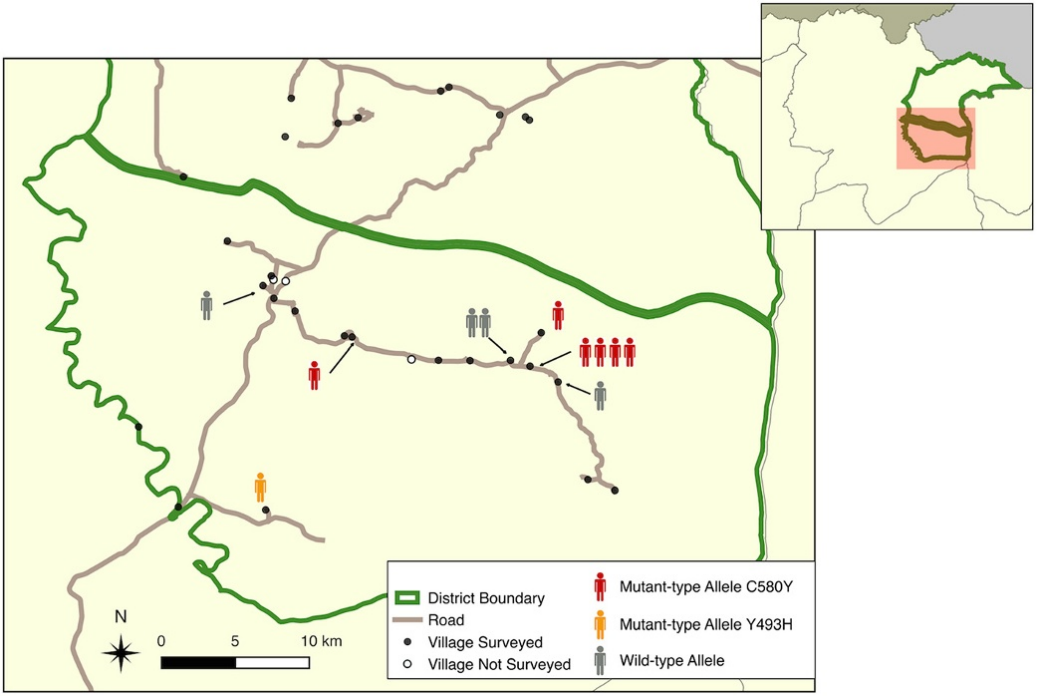
**Geographic distribution of the residence of participants presenting mutations in the K13-propeller domain, Chey Saen district, September – October 2013, Cambodia.**

Of the 27 *P. falciparum* samples, only 11 samples provided sufficient K13 PCR products for sequencing. All were residents of Chey Saen district (Figure 3). The wild-type allele was found in four samples and mutant-type alleles in seven (63%) (C580Y, n = 6 and Y493H, n = 1).

### Malaria prevention measures: mosquito nets

In both Chhaeb (n = 292) and Chey Saen district (n = 285), all participating households owned at least one bed net (median: 2, range: 1–5). In 51.6% of Chhaeb’s and 43.5% of Chey Saen’s households, there was at least one bed net per two individuals. Similar high proportions of participants reported to have slept under a bed net the night preceding the survey: 98.6% in Chhaeb and 97% in Chey Saen.

## Discussion

The primary objectives of this study were to estimate the prevalence of *Plasmodium* spp. and of *P. falciparum* carriers in Chhaeb and Chey Saen districts, and to document the potential presence of artemisinin-resistant parasites. In 2007, the prevalence of *Plasmodium* spp. in Preah Vihear Province was estimated at 12.3% using microscopy [8]. As many *Plasmodium* infections are submicroscopic [18, 19], more sensitive molecular methods were used instead of microscopy. Despite the fact that PCR is more sensitive than microscopy, the 2013 results suggest a considerable decrease of *Plasmodium* spp prevalence since 2007. The observed PCR-based prevalence was similar to the findings of studies conducted in 2010 in Pailin Province (western Cambodia) and in 2012 in Ratanakiri Province (eastern Cambodia). Although these studies were not designed for estimating prevalence at the district level, they used the same PCR approach to detect *Plasmodium* carriage. In Pailin, 1.9% (133/6,931) samples were positive for *Plasmodium spp.*, 45.1% (60) of the species being *P. falciparum* [15] and in Ratanakiri, these figures were respectively 4.9% (244/4,999) and 45.9% [16].

A major concern in prevalence studies designed to guide elimination initiatives is whether all parasite carriers are being captured. While our methodology in this study was robust, we may have underestimated the true prevalence of carriage at two levels. First, we assessed *Plasmodium* spp. carriage by PCR on 5 μl dried blood spots. Compared to higher blood volumes, which would require venipuncture, the collection of 5 μl capillary dried blood spot has undeniable practical advantages (e.g. higher through-put, no cold chain). However, although molecular methods are known to be more sensitive for detection of *Plasmodium* parasites than microscopy [20], the blood volume necessary to maximize the PCR detection of carriers is unclear. Screening higher blood volumes by PCR might allow detecting more carriers, most likely those with lower parasitaemia. Secondly, absentees may also have led to an underestimation of the prevalence. Most absentees were young adult males who were occupied in the forest or in the rice fields at the time of the study. These absentees, as well as the temporary workers living in the compound of private companies who were not included in this study, could be considered as a "hotpop", *i.e*. a population at higher risk of infections due to their occupational behavior [8, 21, 22]. Taking both factors into account, the results likely reflect an underestimation of the prevalence of *Plasmodium* in these districts.

This study illustrated the feasibility of performing a prevalence study relying on molecular methods at a district level. The methodology was not designed to assess the presence of malaria foci, which fell outside of the study objectives. Identifying the hotspots would help to understand the dynamics of transmission in such a low prevalence setting, but requires a different methodology that needs to capture both symptomatic and asymptomatic infections [23]. Indeed, all infected individuals, symptomatic or not, are potentially infectious and contribute to sustain transmission although their exact contribution still has to be evaluated [10, 20, 24]. Most *P. falciparum* and *P. vivax* infections found in this study did not present with fever or history of fever, which is not unusual in low malaria transmission settings [15, 25]. Many infections are submicroscopic [18, 19] and should be detected by appropriate molecular methods. In this study microscopy was not performed and therefore the amount of submicroscopic infections amongst the PCR positive cases is unknown.

The prevalence of *P. falciparum* appeared higher in Chey Saen and *P.vivax* accounted for the majority of infections in Chhaeb. Although numbers were small and there was no statistical evidence for a difference in *P. vivax* prevalence, species preponderance tended to vary across two districts that apparently benefited from similar access to healthcare and malaria control measures. Such possible repartition, potentially the consequence of geographic or socio-demographic factors, needs to be further documented and analysed.

The screening of *P. falciparum* positive samples for mutations in the K13-propeller domain gene suggested the presence of artemisinin-resistant *P. falciparum* in the north of Cambodia, in an area beyond the ‘classical’ western Thai-Cambodian border. Up to now, assessment of artemisinin resistance was performed through strenuous clinical studies [26]. Although the involvement of other genetic modifications cannot be excluded [27], the identification of this molecular marker is a huge step towards possible large-scale surveillance and mapping of artemisinin resistance in Asia and other parts of the world. It is currently not known if resistance has been spreading from another resistant area or if it has emerged *de novo* in the district, but future developments in malaria molecular epidemiology will hopefully help understanding the spread of resistance.

Amongst the 27 *P. falciparum* positive samples of this survey, only 11 could be screened for mutations. In low to very low transmission settings, identifying *P. falciparum* carriers and obtaining sufficient genetic material may become increasingly difficult and collection of higher blood volumes could be considered. In the perspective of monitoring artemisinin resistance using molecular markers, it will be crucial to optimize these techniques and to understand their inherent limits. The two *P. falciparum* detected in Chhaeb district could not be screened, hence the presence of artemisinin resistance in Chhaeb cannot be confirmed. In contrast, in Chey Saen, two thirds of *P. falciparum* screened presented the mutant-type allele and this suggests artemisinin-resistance as being a public health threat in this district.

Consequently, despite the low prevalence of *P. falciparum*, there is little doubt left for the need to intervene and attempt to eliminate the artemisinin-resistant *P. falciparum* in Chey Saen district. The control activities launched in the area might have helped to decrease the level of transmission. However, there is still space for improvement as demonstrated by the unsatisfactory mosquito nets ownership in this study, with only half of the households owning a net per two persons. Unfortunately, there is no standard solution for elimination and, from a public health perspective, the challenges are numerous. Interventions need to target the hotspots of transmission, the high risk populations (the "hotpops", which need to be defined better), as well as the mobile populations, because imported cases may be an important source of infection [21]. To identify asymptomatic carriers, very sensitive PCR-based screening methods are needed and the choice of these methods might be guided by further research on the contribution of low parasitaemic carriers to transmission [19]. Several interventions have been proposed, such as active case detection (ACD), mass screening and treatment (MSaT), focused or focal screening and treatment (FSaT), and mass drug administration (MDA) [28]. Despite some interesting feedback from field experiences, their effectiveness in various transmission settings has yet to be validated [21].

Developing methods to assess the impact of these interventions is challenging. As *Plasmodium* carriage will further decrease, an adequate sample size and study power will become more difficult to reach [29, 30]. Considering the limits of existing indicators in low transmission settings (annual parasite incidence, annual case incidence, entomological inoculation rate, parasite rate) [29], new monitoring and evaluation tools are also needed, based on sensitive indicators to track progresses towards elimination [31]. Serology might become an indicator of choice in the future [29, 32, 33] but before being used for routine monitoring of transmission, reagents, methodologies and protocols still need to be standardized [20].

Finally, the interest of the community in joining the fight towards elimination is a cornerstone of any intervention. During our study, villages’ key-informants expressed malaria as a waning concern nowadays, representing only a minor cause of deaths. Regardless of the type of intervention, specific attention should be given to community involvement, otherwise all efforts would be jeopardized [34].

## Conclusions

The overall prevalence of *Plasmodium* spp. and *P. falciparum* was low in both districts, indicating that activities aiming at malaria elimination should complement existing malaria control measures in these settings. Mutations in the K13-propeller domain gene, suggestive for artemisinin-resistance were observed in *Plasmodium falciparum* parasites in Chey Saen district, despite important containment efforts conducted in the last years. This underlines the urgent need to reinforce current control measures and to define and implement innovative interventions to eliminate the *P. falciparum* reservoir from this area, from similar zones in Cambodia and neigbhouring countries, before its spread to other malaria endemic zones.

## References

[1] WHO (2013). World Malaria Report 2013.

[2] Delacollette C, D’Souza C, Christophel E, Thimasarn K, Abdur R, Bell D, Dai TC, Gopinath D, Lu S, Mendoza R, Ortega L, Rastogi R, Tantinimitkul C, Ehrenberg J (2009). Malaria trends and challenges in the Greater Mekong Subregion. Southeast Asian J Trop Med Public Health.

[3] Noedl H, Se Y, Schaecher K, Smith BL, Socheat D, Fukuda MM (2008). Evidence of Artemisinin-Resistant Malaria in Western Cambodia. N Engl J Med.

[4] Dondorp AM, Nosten F, Yi P, Das D, Phyo AP, Tarning J, Lwin KM, Ariey F, Hanpithakpong W, Lee SJ, Ringwald P, Silamut K, Imwong M, Chotivanich K, Lim P, Herdman T, An SS, Yeung S, Singhasivanon P, Day NP, Lindegardh N, Socheat D, White NJ (2009). Artemisinin resistance in *Plasmodium falciparum* malaria. N Engl J Med.

[5] Amaratunga C, Sreng S, Suon S, Phelps ES, Stepniewska K, Lim P, Zhou C, Mao S, Anderson JM, Lindegardh N, Jiang H, Song J, Su X, White NJ, Dondorp AM, Anderson TJC, Fay MP, Mu J, Duong S, Fairhurst RM (2012). Artemisinin-resistant *Plasmodium falciparum* in Pursat province, western Cambodia: a parasite clearance rate study. Lancet Infect Dis.

[6] WHO (2010). Global Report on Antimalarial Drug Efficacy and Drug Resistance: 2000–2010.

[7] WHO (2011). Global plan for artemisinin resistance containment (GPARC).

[8] Incardona S, Vong S, Chiv L, Lim P, Nhem S, Sem R, Khim N, Doung S, Mercereau-Puijalon O, Fandeur T (2007). Large-scale malaria survey in Cambodia: novel insights on species distribution and risk factors. Malar J.

[9] ACTwatch Group and PSI/Cambodia (2011). Kingdom of Cambodia Household Survey Report, 2011.

[10] Okell LC, Bousema T, Griffin JT, Ouédraogo AL, Ghani AC, Drakeley CJ (2012). Factors determining the occurrence of submicroscopic malaria infections and their relevance for control. Nat Commun.

[11] Steenkeste N, Incardona S, Chy S, Duval L, Ekala M-T, Lim P, Hewitt S, Sochantha T, Socheat D, Rogier C, Mercereau-Puijalon O, Fandeur T, Ariey F (2009). Towards high-throughput molecular detection of Plasmodium: new approaches and molecular markers. Malar J.

[12] Toma H, Kobayashi J, Vannachone B, Arakawa T, Sato Y, Nambanya S, Manivong K, Inthakone S (2001). A field study on malaria prevalence in southeastern Laos by polymerase chain reaction assay. Am J Trop Med Hyg.

[13] Ariey F, Witkowski B, Amaratunga C, Beghain J, Langlois A-C, Khim N, Kim S, Duru V, Bouchier C, Ma L, Lim P, Leang R, Duong S, Sreng S, Suon S, Chuor CM, Bout DM, Ménard S, Rogers WO, Genton B, Fandeur T, Miotto O, Ringwald P, Le Bras J, Berry A, Barale J-C, Fairhurst RM, Benoit-Vical F, Mercereau-Puijalon O, Ménard D (2014). A molecular marker of artemisinin-resistant *Plasmodium falciparum* malaria. Nature.

[14] **Commune Database Online (for Cambodia)** http://db.ncdd.gov.kh/cdbonline/home/index.castle

[15] Hoyer S, Nguon S, Kim S, Habib N, Khim N, Sum S, Christophel E-M, Bjorge S, Thomson A, Kheng S, Chea N, Yok S, Top S, Ros S, Sophal U, Thompson MM, Mellor S, Ariey F, Witkowski B, Yeang C, Yeung S, Duong S, Newman RD, Menard D (2012). Focused screening and treatment (FSAT): a PCR-based strategy to detect malaria parasite carriers and contain drug resistant P. falciparum, Pailin, Cambodia. PLoS One.

[16] Canier L, Khim N, Kim S, Sluydts V, Heng S, Dourng D, Eam R, Chy S, Khean C, Loch K, Ken M, Lim H, Siv S, Tho S, Masse-Navette P, Gryseels C, Uk S, Van Roey K, Grietens KP, Sokny M, Thavrin B, Chuor CM, Deubel V, Durnez L, Coosemans M, Ménard D (2013). An innovative tool for moving malaria PCR detection of parasite reservoir into the field. Malar J.

[17] Menard D, Ariey F (2013). PCR_Sequencing for genotyping SNPs PF3D7_1343700 Kelch protein propeller domain. Protoc Exch.

[18] Steenkeste N, Rogers WO, Okell L, Jeanne I, Incardona S, Duval L, Chy S, Hewitt S, Chou M, Socheat D, Babin F-X, Ariey F, Rogier C (2010). Sub-microscopic malaria cases and mixed malaria infection in a remote area of high malaria endemicity in Rattanakiri province, Cambodia: implication for malaria elimination. Malar J.

[19] Lin JT, Saunders DL, Meshnick SR (2014). The role of submicroscopic parasitemia in malaria transmission: what is the evidence?. Trends Parasitol.

[20] World Health Organization (2014). WHO Evidence Review Group on Malaria Diagnosis in Low Transmission Settings.

[21] Sturrock HJW, Hsiang MS, Cohen JM, Smith DL, Greenhouse B, Bousema T, Gosling RD (2013). Targeting asymptomatic malaria infections: active surveillance in control and elimination. PLoS Med.

[22] Cotter C, Sturrock HJW, Hsiang MS, Liu J, Phillips A a, Hwang J, Gueye CS, Fullman N, Gosling RD, Feachem RG (2013). The changing epidemiology of malaria elimination: new strategies for new challenges. Lancet.

[23] Bousema T, Griffin JT, Sauerwein RW, Smith DL, Churcher TS, Takken W, Ghani A, Drakeley C, Gosling R (2012). Hitting hotspots: spatial targeting of malaria for control and elimination. PLoS Med.

[24] Laishram DD, Sutton PL, Nanda N, Sharma VL, Sobti RC, Carlton JM, Joshi H (2012). The complexities of malaria disease manifestations with a focus on asymptomatic malaria. Malar J.

[25] Harris I, Sharrock WW, Bain LM, Gray K-A, Bobogare A, Boaz L, Lilley K, Krause D, Vallely A, Johnson M-L, Gatton ML, Shanks GD, Cheng Q (2010). A large proportion of asymptomatic Plasmodium infections with low and sub-microscopic parasite densities in the low transmission setting of Temotu Province, Solomon Islands: challenges for malaria diagnostics in an elimination setting. Malar J.

[26] Flegg JA, Guerin PJ, White NJ, Stepniewska K (2011). Standardizing the measurement of parasite clearance in falciparum malaria: the parasite clearance estimator. Malar J.

[27] White NJ (2014). Malaria: a molecular marker of artemisinin resistance. Lancet.

[28] WHO (2013). Consideration of Mass Drug Administration for the Containment of Artemisinin Resistant Malaria in the Greater Mekong Subregion.

[29] The malERA Consultative Group on Monitoring Evaluation and Surveillance (2011). A research agenda for malaria eradication: monitoring, evaluation, and surveillance. PLoS Med.

[30] Stresman G, Kobayashi T, Kamanga A, Thuma PE, Mharakurwa S, Moss WJ, Shiff C (2012). Malaria research challenges in low prevalence settings. Malar J.

[31] The malERA Consultative Group on Integration Strategies (2011). A research agenda for malaria eradication: cross-cutting issues for eradication. PLoS Med.

[32] Rosas-Aguirre A, Llanos-Cuentas A, Speybroeck N, Cook J, Contreras-Mancilla J, Soto V, Gamboa D, Pozo E, Ponce OJ, Pereira MO, Soares IS, Theisen M, D’Alessandro U, Erhart A (2013). Assessing malaria transmission in a low endemicity area of north-western Peru. Malar J.

[33] Hsiang MS, Hwang J, Kunene S, Drakeley C, Kandula D, Novotny J, Parizo J, Jensen T, Tong M, Kemere J, Dlamini S, Moonen B, Angov E, Dutta S, Ockenhouse C, Dorsey G, Greenhouse B (2012). Surveillance for malaria elimination in Swaziland: a national cross-sectional study using pooled PCR and serology. PLoS One.

[34] Atkinson J-A, Johnson M-L, Wijesinghe R, Bobogare A, Losi L, O’Sullivan M, Yamaguchi Y, Kenilorea G, Vallely A, Cheng Q, Ebringer A, Bain L, Gray K, Harris I, Whittaker M, Reid H, Clements A, Shanks D (2012). Operational research to inform a sub-national surveillance intervention for malaria elimination in Solomon Islands. Malar J.

